# Design and Analysis of an O+E-Band Hybrid Optical Amplifier for CWDM Systems

**DOI:** 10.3390/mi13111962

**Published:** 2022-11-12

**Authors:** Benish Kanwal, Ammar Armghan, Salman Ghafoor, Ahmad Atieh, Muhammad Sajid, Tasleem Kausar, Jawad Mirza, Yun Lu

**Affiliations:** 1 Electrical Engineering Department, Mirpur University of Science and Technology, Mirpur 10250, Pakistan; 2 Department of Electrical Engineering, College of Engineering, Jouf University, Sakaka 72388, Saudi Arabia; 3 School of Electrical Engineering and Computer Science, National University of Sciences and Technology, Islamabad 44000, Pakistan; 4 Optiwave Systems Inc., Ottawa, ON K1N 6N5, Canada; 5 SEECS Photonics Research Group, Islamabad 44000, Pakistan; 6 School of Computer Science and Engineering, Huizhou University, Huizhou 516007, China

**Keywords:** hybrid optical amplifier, O+E band amplifier, praseodymium-doped fiber amplifier, semiconductor optical amplifier

## Abstract

Broadband amplification in the O+E-band is very desirable nowadays as a way of coping with increasing bandwidth demands. The main issue with doped fiber amplifiers working in this band such as the bismuth-doped fiber amplifier is that they are costly and not widely available. Therefore, a wideband and flat-gain hybrid optical amplifier (HOA) covering the O+E-band based on a parallel combination of a praseodymium-doped fiber amplifier (PDFA) and a semiconductor optical amplifier (SOA) is proposed and demonstrated through numerical simulations. The praseodymium-doped fiber (PDF) core is pumped using a laser diode with a power of 500 mW that is centered at a wavelength of 1030 nm. The SOA is driven by an injection current of 60 mA. The performance of the HOA is analyzed by the optimization of various parameters such as the PDF length, Pr${}^{3+}$ concentration, pump wavelength, and injection current. A flat average gain of 24 dB with a flatness of 1 dB and an output power of 9.6 dBm is observed over a wavelength range of 1270–1450 nm. The noise figure (NF) varies from a minimum of 4 dB to a maximum of 5.9 dB for a signal power of 0 dBm. A gain reduction of around 4 dB is observed for an O-band signal at a wavelength of 1290 nm by considering the up-conversion effect. The transmission performance of the designed HOA as a pre-amplifier is evaluated based on the bit-error rate (BER) analysis for a coarse wavelength-division multiplexing (CWDM) system of eight on-off keying (OOK)-modulated channels, each having a data rate of 10 Gbps. An error-free transmission over 60 km of standard single-mode fiber (SMF) is achieved for different data rates of 5 Gbps, 7.5 Gbps, and 10 Gbps.

## 1. Introduction

IP traffic is continuously increasing globally due to the proliferation of various applications and technologies in our daily lives such as fifth-generation mobile networks, cloud computing, web applications, and the Internet of Things (IoT) [1,2]. The tendency of increased bandwidth demands and, therefore, network capacity will persist in the future. Urgent steps are required to meet the enormous bandwidth demands since the commonly employed C-band for commercial optical links is facing its capacity limits [2]. Fiber-optic communication is mainly conducted in the wavelength region where optical fibers have relatively low transmission loss [2]. This low-loss wavelength region ranges from 1260 nm to 1650 nm and is divided into six wavelength bands referred to as the O-, E-, S-, C-, L-, and U-bands, which range from 1260–1360 nm, 1360–1460 nm, 1460–1530 nm, 1530–1565 nm, 1565–1625 nm, and 1625–1675 nm, respectively [2]. Moreover, the O-band has smaller fiber dispersion, which enables high-speed optical transmission in this band without using any dispersion compensation schemes [3]. Similarly, the E-band has had a small transmission loss compared to the O-band since the invention of dehydration techniques during glass production [3]. Different solutions are available to fix the above-mentioned capacity limits of optical communication systems, which include (a) the optimal use of advanced multi-level modulation formats, (b) spatial division multiplexing (SDM)-based systems, and (c) exploiting the relatively low attenuation and unused optical windows for transmission beyond the C-band [2,4]. The first two approaches can be considered for addressing the issues of capacity and bandwidth demand at the expense of certain issues, which have been discussed in [2,4]. The third approach that exploits the high bandwidth provided by SMFs is generally called optical multiband transmission (OMBT) and is the most viable and efficient solution [4]. The OMBT technique relies on the use of the low-attenuation optical bands of SMF for data transmission, resulting in an 11-fold expansion of the available bandwidth of the C-band and 5 times the available bandwidth of the C+L-band [5]. The first phase of the OMBT scheme targets the implementation of communication outside the C-band such as the L-band using commercial off-the-shelf components [5]. In the second phase, the remaining bands, such as the O-, E-, S-, and U-bands, will be considered for data transmission [5]. The upgrade to the L-band is carried out based on erbium-doped fiber amplifiers (EDFAs), thus enabling the addition of a massive bandwidth of around 60 nm to the current 35 nm window of the C-band [2,5]. Therefore, the main challenge for the realization of OMBT for the second phase is the design and realization of innovative, efficient, and low-cost photonic components, particularly optical sources and wideband amplifiers compatible with the O-, E-, S-, and U-bands.

In optical fiber communication, optical signals suffer from various impairments such as fiber attenuation, component insertion losses, fiber dispersion, and nonlinearities [6]. The capacity of optical networks can be increased by providing a sufficient power budget or by increasing the signal power through the use of amplifiers at regular intervals along the fiber link [7,8]. Initially, electronics-based semiconductor amplifiers were employed to boost the power of optical signals. However, these amplifiers had some issues such as the requirement of optical-to-electrical and then electrical-to-optical conversions, low reliability, complexity, bulkiness, and cost inefficiency [9]. All-optical fiber amplifiers have been extensively researched and various doped fiber amplifiers based on the rare-earth dopants ytterbium, praseodymium, thulium, and holmium have been proposed [6].

Another way to realize wideband optical amplifiers is to combine multiple optical amplifiers in parallel or in series, where each amplifier operates over a distinct spectral region [10]. Such an arrangement is generally known as a hybrid optical amplifier (HOA) and has been shown to enhance system capacity and performance [11]. HOAs can be realized using different combinations of series or parallel amplifiers, as discussed in [12]. Alabbas et al. proposed a C+L-band HOA based on hafnia–bismuth–erbium-doped fiber (HBEDF) and zirconia–erbium-doped fiber (ZEDF) as the gain medium [13]. For the design proposed in [13], a gain of 14.6 dB, gain fluctuation of 1.8 dB, and NF fluctuating in the range of 4.3–7.9 dB over a wavelength range of 1530–1600 nm was observed. HOAs that have been demonstrated in other studies include the HOA based on distributed Raman-EDFA that was used for amplifying a 120 Tbps optical signal over 630 km of SMF [14], Raman-EDFA used for a 54 Tbps optical signal over 9150 km of SMF [15], and Raman-SOA that was used for a 107 Tbps optical signal over 300 km of SMF [16]. Guo et al. proposed an HOA based on a combination of two fiber parametric wavelength converters and an EDFA for S-band amplification [17]. An NF as low as 4 dB was observed when the conversion efficiency was kept higher than 10 dB for the first stage. Kaur et al. designed an HOA based on SOA-EDFA-Raman for the transmission of 40 DWDM channels at a rate of 10 Gbps over 240 km of SMF operating at the edge of the L- and U-bands [18]. A gain of 31 dB with a flatness of 0.8 dB and an NF of 5.7 dB was observed for a 1611.8–1620.5 nm wavelength range. Hafiz et al. demonstrated an erbium–ytterbium co-doped waveguide amplifier (EYDWA)-Raman HOA having a gain of 25 dB with a flatness of 2.78 dB and an NF of less than 6 dB over a 1539.7–1562.7 nm wavelength range [19]. Hafiz et al. proposed an HOA based on an erbium–ytterbium co-doped fiber amplifier (EYDFA) and backpropagating Raman amplifier [20]. A gain of 26 dB was achieved with a flatness of 1.37 dB over a wavelength range of 1545–1565 nm. In [21], an HOA based on a Raman-EDFA configuration was proposed for WDM transmissions. The HOA exhibited a gain of 46 dB and an NF of 3 dB over a 1530–1600 nm wavelength range. R.E.Tench reported designs of HOAs based on a three-stage holmium-doped fiber amplifier (HDFA) and a thulium-doped fiber amplifier (TDFA) [22] and a two-stage HDFA and TDFA employing a shared pump [23]. A small signal gain of 70 dB over a 2009-2098 nm wavelength range and an NF of 7.5 dB [22], 49.1 dB, and 6.5 dB at 2051 nm [23] were observed. Maes et al. reported on an E+S-band HOA based on bismuth-doped fiber (BDF) and EDF, which produced a small signal gain and output power of 27 dB and 24.5 dBm, respectively over a 1431–1521 nm wavelength range [24]. Guo et al. demonstrated a three-stage S-band HOA based on an L-band EDFA placed between two optical parametric amplifier (OPA)-based wavelength converters [25]. An average gain of 18.6 dB with a flatness of 1.2 dB and an NF of 5.1 dB was observed. F. D. Ros et al. optimized a C+L-band HOA based on an EDFA-Raman amplifier using neural network models, where gain flatness decreased from 6.7 dB to 1.9 dB [26]. A survey of the previous work related to HOAs has been summarized in Table 1 and compared with the main results obtained from our proposed work. The missing information in the studies presented in Table 1 is represented by dashes.

In this paper, we proposed a wideband flat-gain O+E-band HOA based on a parallel configuration of PDFA-SOA. Wideband fiber Bragg grating (WFBG) has been used to separate the O- and E-band signals to input them separately into the PDFA and the SOA, respectively. In our previous study, we optimized the values of the doping concentration of Pr${}^{3+}$ and the length of the PDF [6,27]. In this work, we optimized the pump wavelength and the injection current to analyze the performance of the proposed HOA. A small signal gain of 24 dB with a flatness of around 1 dB and an NF of 4–5.9 dB over a 1270–1450 nm wavelength range was observed. Finally, the transmission performance of the HOA was evaluated as a pre-amplifier in a CWDM system of eight OOK-modulated optical signals over 60 km of SMF and an aggregate data rate of 80 Gbps. We implemented the proposed HOA using the well-known commercial tool called OptiSystem [28]. The proposed HOA can be used to enable amplification in future optical access networks.

## 2. Theoretical Background

### Spectroscopic Properties and Rate Equations of Pr${}^{3+}$

**Figure 1:**
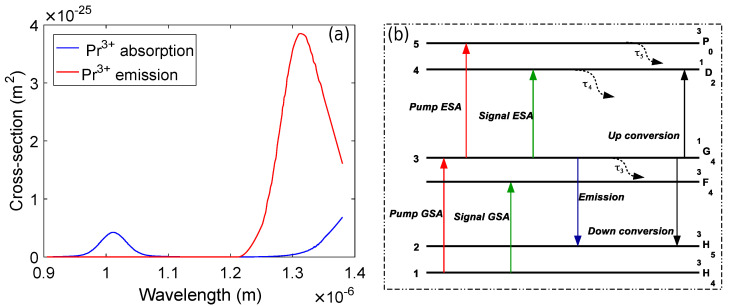
(**a**) Pr${}^{3+}$ absorption and emission spectra. (**b**) Energy level diagram.

Figure 1 exhibits the absorption and emission cross-sections and the simplified energy level diagram of Pr${}^{3+}$.

It is evident from Figure 1a that Pr${}^{3+}$ has two pump absorption bands with their peaks centered at 1010 nm and 1400 nm. The pump wavelengths in the 1000–1040 nm wavelength range are widely used to excite the Pr${}^{3+}$ [6,27]. The emission starts at 1220 nm but the emission cross-section is maximum at around 1300 nm. The simplified energy level diagram of Pr${}^{3+}$ based on four-level absorption and radiative transitions is shown in Figure 1b. It can be observed that the main energy levels are labeled as ^1^G_4_, ^3^P_0_, ^1^D_0_, ^3^F_4_, ^3^F_3_, ^3^H_5_, and ^3^H_4_ [29]. The ^1^G_4_↔^3^H_4_ transitions enable the pump ground state absorption (GSA) and emission. Similarly, the ^3^H_4_→^3^F_4_ and ^1^G_4_→^3^H_5_ transitions hold the signal GSA and signal emission. Moreover, the carrier density of the ^1^G_4_ level can be decreased by the up-conversion effect due to the ^1^G_4_→^1^D_2_ and ^1^G_4_→^3^H_5_ transitions [29] because the energy difference between the ^1^G_4_ and ^1^D_2_ levels is equal to the energy difference between the ^1^G_4_ and ^3^H_5_ levels [6,27,29]. The carrier densities at each level are represented by $n_{1}$, $n_{2}$, $n_{3}$, $n_{4}$, and $n_{5}$ and the total density $n_{t}$ is given by [27]:

(1) $$n_{t}=n_{1}+n_{2}+n_{3}+n_{4}+n_{5}$$

The rate equations for the energy level diagram of Pr${}^{3+}$ shown in Figure 1b can be written as [27]:

(2) $$\frac{dn_{3}}{dt}=\gamma_{13}n_{1}-\left(\gamma_{35}+\gamma_{34}+\gamma_{32}+\gamma_{31}+\frac{1}{\tau_{3}}+cn_{3}\right)n_{3}+\frac{B_{43}}{\tau_{4}}n_{4}+\frac{B_{53}}{\tau_{5}}n_{5}$$

(3) $$\frac{dn_{4}}{dt}=\left(\gamma_{35}+\frac{c}{2}n_{3}\right)n_{3}-\frac{n_{4}}{\tau_{4}}$$

(4) $$\frac{dn_{5}}{dt}=\gamma_{35}n_{3}-\frac{n_{5}}{\tau_{5}}$$

In the above expressions, the transition rates $\gamma_{13}$, $\gamma_{31}$, $\gamma_{32}$, $\gamma_{34}$, and $\gamma_{35}$ are given by $\frac{P_{p}\sigma_{13}\eta_{p}}{A_{c}h\upsilon_{p}}$, $\frac{P_{p}\sigma_{31}\eta_{p}}{A_{c}h\upsilon_{p}}$, $\frac{P_{s}\sigma_{32}\eta_{s}}{A_{c}h\upsilon_{s}}$, $\frac{P_{s}\sigma_{34}\eta_{s}}{A_{c}h\upsilon_{s}}$, and $\frac{P_{s}\sigma_{35}\eta_{s}}{A_{c}h\upsilon_{s}}$, respectively. Similarly, $B_{53}$ and $B_{43}$ are the branching ratios for the ^3^P_0_–^1^G_4_ and ^1^D_2_–^1^G_4_ transitions, which are evaluated by the Judd–Ofelt analysis as having numerical values of 2 % and 9 %, respectively.

The small-signal gain of the PDFA completely depends upon the transitions between the ^3^H_4_ and ^1^G_4_ levels having carrier densities of *n*${}_{1}$ and *n*${}_{3}$, respectively. When a signal passes through the gain medium of the PDFA having a thickness of dz, then the propagation equation of the signal can be written as [27,29]:

(5) $$\frac{dP_{s}}{dz}=\left[n_{3}\left(\sigma_{32}-\sigma_{34}\right)-n_{1}\sigma_{13}\right]P_{s}\Gamma_{s}$$

In the above expression, $\sigma_{34}$ is the excited state absorption (ESA) cross-section at the signal wavelength, which is not considered by OptiSystem. Therefore, the small-signal gain achieved is given by [27,29]:

(6) $$G=\frac{P_{s}\left(L\right)}{P_{s}\left(0\right)}=exp\left[L\Gamma_{s}\left\{n_{3}\left(\sigma_{32}-\sigma_{34}\right)-n_{1}\sigma_{13}\right\}\right],$$

where *L* is the length of the PDF.

The OptiSystem model of traveling-wave SOA performs lumped amplification, which is applicable to describe the amplification of both the CW and the optical pulsed signals. The coefficient of material gain $g_{m}$ and carrier density N(t) are interrelated by the expression [30]

(7) $$g_{m}\left(t\right)=A_{g}\left[N\left(t\right)-N_{o}\right]$$

Similarly, the net gain coefficient *g* and material gain $g_{m}$ are also related to each other by [30]

(8) $$g\left(t\right)=\Gamma g_{m}\left(t\right)-\alpha$$

Neglecting the group velocity dispersion (GVD) in the SOA and taking into account the amplified spontaneous emission (ASE), the gain *G* for a traveling-wave SOA at a distance *z* is given by [30]

(9) $$G\left(t,z\right)=exp\left[g(t)z\right]$$

The carrier and optical intensities are related by the following rate equation [30]:

(10) $$\frac{dn}{dt}=\frac{j}{ed}-R\left(n\right)-\frac{\Gamma g_{m}}{E}\left(\beta I_{sp}+I\right),$$

where β is the spontaneous emission coefficient, which characterizes the part of the total spontaneous emission coupled to the guided wave and is given by [30]:

(11) $$\beta =\frac{c}{N_{m}}\frac{a\tau }{V}$$

The recombination rate is generally assumed to be linearly proportional to the carrier density in order to obtain almost accurate results; therefore, $R\left(n\right)=\frac{n}{r}$. Assuming α=0 [30], the steady-state expression becomes

(12) $$\frac{j}{ed}=\frac{n}{\tau }+\frac{\Gamma g_{m}}{E}I$$

The various symbols used in Equations (1)–(12) are described in Table 2.

## 3. Proposed Design of Hybrid Optical Amplifier

**Figure 2:**
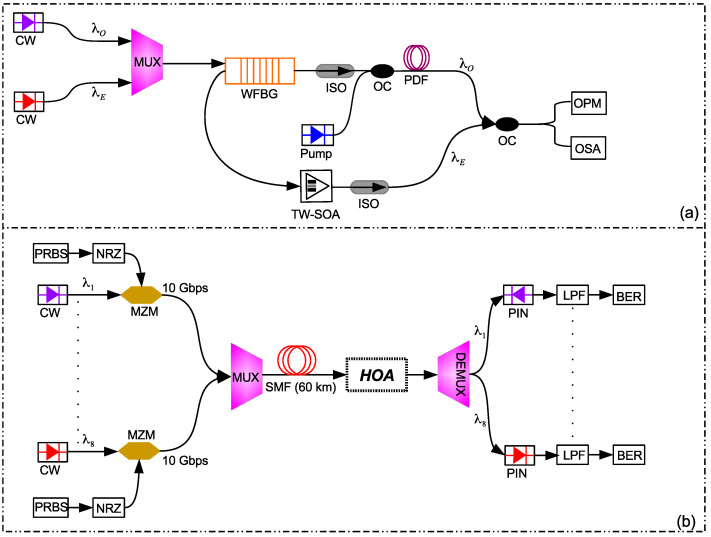
Schematic of (**a**) the proposed HOA and (**b**) the system-level integration of the HOA in an eight-channel CWDM transmission system.

Figure 2a shows the schematic of the proposed design of an HOA for O+E-band amplification based on a parallel combination of a PDFA and an SOA. Figure 2b illustrates the implementation of the proposed HOA in a typical eight-channel CWDM transmission link. The HOA consists of a wavelength division multiplexer (WDM) used to combine the O- and E-band signals, a reflective-type WFBG filter used to separate the O- and E-band wavelengths for amplification, a laser diode pump, and two optical isolators used to block any light reflected back into the system. The O- and E-band wavelengths are combined using a WDM coupler and then applied to the WFBG, whose center wavelength is adjusted to 1310 nm. The WFBG reflects all E-band signals and transmits all O-band signals, as shown in Figure 2. The O-band signal is coupled with a pump laser with a wavelength and power of 1030 nm and 500 mW, respectively, and injected into the PDF. Similarly, the E-band signal is applied to the input of the SOA for amplification. The injection current of the SOA is adjusted such that the gain fluctuation in the E-band is minimum. The injection current used to drive the SOA in this work is 60 mA. The O- and E-band signals are combined after amplification using an optical coupler. A two-port WDM analyzer, an optical power meter (OPM), and an optical spectrum analyzer (OSA) are employed in the simulation for the observation and analysis of the results.

Figure 2b shows eight CW lasers centered at wavelengths of $\lambda_{1}=1270$ nm, $\lambda_{2}=1290$ nm, $\lambda_{3}=1310$ nm, $\lambda_{4}=1370$ nm, $\lambda_{5}=1390$ nm, $\lambda_{6}=1410$ nm, $\lambda_{7}=1430$ nm, and $\lambda_{8}=1450$ nm, each modulated by non-return-to-zero (NRZ) data at a rate of 10 Gbps. A pseudo-random bit-sequence generator (PRBS) inputs the logical data to the NRZ generator that converts the data into an electrical signal. After modulation with a Mach–Zehnder modulator (MZM), the resultant optical signals are multiplexed (MUX) and transmitted over a standard SMF of 60 km. The combined 80 Gbps CWDM signal is amplified using the proposed O+E-band HOA. At the receiving end, the optical CWDM signal is demultiplexed (DEMUX). Each of the outputs of the DEMUX is photodetected using a PIN photodiode, low-pass filtered (LPF) to remove the band noise and harmonics, and given to bit-error rate (BER) estimators. Table 3 shows the values of the important parameters used in our study.

## 4. Results and Discussion

**Figure 3:**
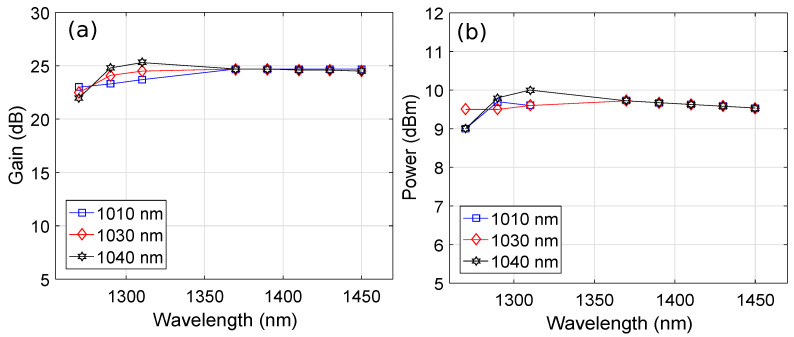
Wavelength versus (**a**) gain plots as a function of the pump wavelength and (**b**) output power plots as a function of the pump wavelength.

The length of the PDF, doping concentration of Pr${}^{3+}$, pump wavelength, and injection current were optimized to achieve the optimum performance of the HOA. As mentioned earlier, we used the previously optimized values of the doping concentration of Pr${}^{3+}$ and PDF length [6,27], whereas the pump wavelength and injection current were optimized in this study. Figure 3 shows the plots of the pump wavelength versus the amplifier gain and output power by considering the PDF length, Pr${}^{3+}$ concentration, and SOA injection current of 15.7 m, $50\times 10^{24}$ m${}^{-3}$, and 60 mA, respectively, at an input signal power of -15 dBm. It is clear from Figure 3a,b that an average gain and output power of around 24 dB and 9.6 dBm were observed, respectively, over a 1270–1450 nm wavelength range covering the O+E-band for a pump wavelength of 1010 nm, 1030 nm, and 1040 nm. It is evident from Figure 3a,b that the flatness in gain and power was best for a pump wavelength of 1030 nm. Therefore, we use a pump wavelength of 1030 nm for the rest of the results.

**Figure 4:**
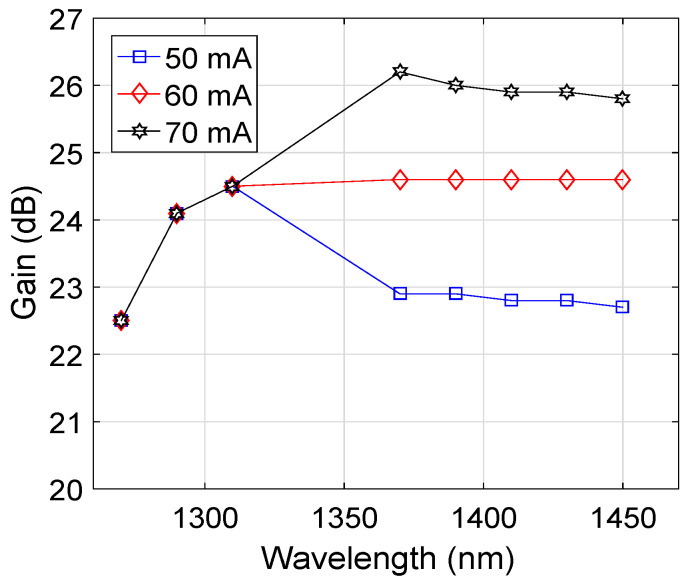
Wavelength versus gain plots as a function of injection current.

Figure 4 shows the wavelength versus gain plots as a function of the injection current of the SOA considering a PDF length of 15.7 m, Pr${}^{3+}$ concentration of $50\times 10^{24}$ m${}^{-3}$, and pump wavelength of 1030 nm for an input signal power of −15 dBm. It may appear that the fluctuation in the gain of the HOA was at a minimum for an injection current of 60 mA, particularly in the E-band, compared to 50 mA and 70 mA. Therefore, 60 mA was used as the optimized value of the injection current of the SOA to obtain an overall flat-gain profile for the HOA. Moreover, it can be observed that the gain of the HOA in the E-band increased by increasing the injection current of the SOA.

**Figure 5:**
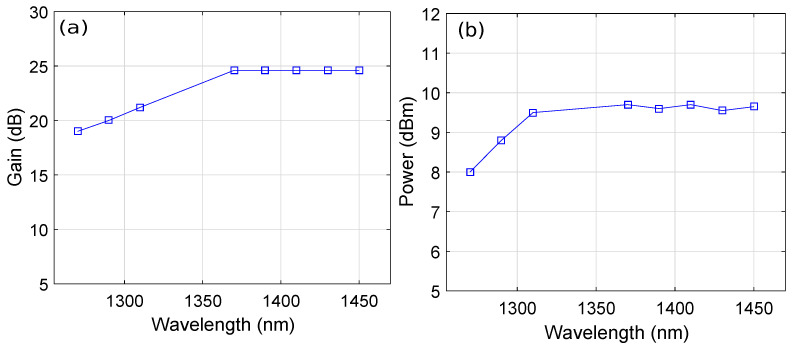
Wavelength versus (**a**) gain plots considering the up-conversion effect and (**b**) output power plots considering the up-conversion effect.

To validate the impact of up-conversion on the gain and output power of the HOA, we obtained the wavelength versus gain and output power plots for the HOA by using a PDF length of 15.7 m, Pr${}^{3+}$ concentration of $50\times 10^{24}$ m${}^{-3}$, pump wavelength of 1030 nm, and injection current of 60 mA, respectively. The results are shown in Figure 5 for a signal power of −15 dBm. Figure 5a shows that a penalty of around 4 dB was observed for the gain of the HOA for an O-band wavelength of 1290 nm. Similarly, Figure 5b shows that a penalty of around 1 dB was observed in the output power of the HOA for an O-band wavelength of 1290 nm. These results confirm that up-conversion had a negative impact on the gain and output power of the PDFA owing to the decrement in the population inversion originating when a Pr${}^{3+}$ ion jumped to an excited manifold, whereas the other was demoted to a low-energy manifold [27,31]. It is worth mentioning here that up-conversion occurred only in the PDF, which subsequently affected the wavelengths in the O-band only by reducing the gain and output power, whereas the gain and output power remained constant in the E-band because the phenomenon of up-conversion did not happen in the SOAs.

**Figure 6:**
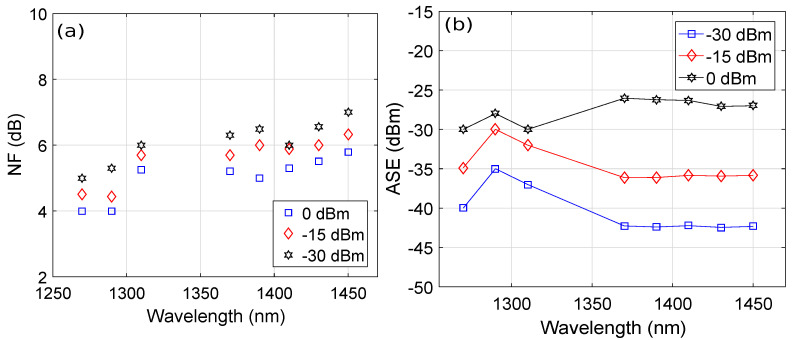
Wavelength versus (**a**) NF plots as a function of signal power and (**b**) ASE plots as a function of signal power.

We also plotted the wavelength versus the NF as a function of the signal power, as shown in Figure 6a, considering a PDF length of 15.7 m, Pr${}^{3+}$ concentration of $50\times 10^{24}$ m${}^{-3}$, pump wavelength of 1030 nm, pump power of 500 mW, and injection current of 60 mA. The results show that the NF was equal to 4 dB, 4.5 dB, and 5 dB at a signal wavelength of 1270 nm for signal powers of 0 dBm, −15 dBm, and −30 dBm, respectively. The NF increased to around 5.3 dB, 5.8 dB, and 6 dB for a signal wavelength of 1410 nm and signal powers of 0 dBm, −15 dBm, and −30 dBm, respectively. Therefore, it is clear that the NF increased when the power of the signal was low. The reason behind this trend is that the optical signal-to-noise ratio (OSNR) was reduced when the signal power was weak, which, in turn, increased the NF of the HOA [6]. We also plotted the signal wavelength versus the ASE as a function of the signal power in Figure 6b by considering a PDF length of 15.7 m, Pr${}^{3+}$ concentration of $50\times 10^{24}$ m${}^{-3}$, pump wavelength of 1030 nm, pump power of 500 mW, and injection current of 60 mA. It can be observed from the spectral plot in Figure 6b that the average ASEs over a 1270–1450 nm wavelength range were around −39 dBm, −33.7 dBm, and −28 dBm for signal powers of −30 dBm, −15 dBm, and 0 dBm, respectively. Moreover, it is also evident that the ASE increased by increasing the signal power. The reason behind this trend is that by increasing the signal power, the photon population due to spontaneous emission relatively increased, which consequently resulted in an increase in the ASE [6].

**Figure 7:**
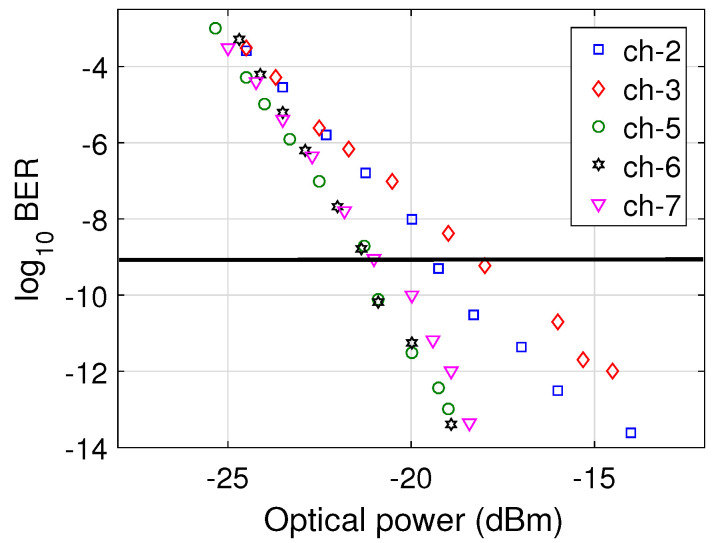
BER versus detected optical power plots of different channels.

To observe the performance of the proposed O+E-band HOA in a transmission link, we employed it in a CWDM system and measured the BER of the signals transmitted over the link. The BER was computed by observing the statistical distribution of the eye diagrams of the received signals obtained after the low-pass filter (LPF) shown in Figure 2b. The optical power received for each channel at the PIN photodetectors was varied by using optical attenuators to observe the impact on the BER values. The minimum detected optical power necessary to obtain a BER of 10${}^{-9}$ is called the receiver sensitivity [32]. Figure 7 shows the BER versus the received optical power plots for channels 2, 3, 5, 6, and 7, where each channel had a data rate of 10 Gbps. We have chosen a limited number of channels to make the results easy to visualize. It is clear that the receiver sensitivities of channels 5, 6, and 7 were around −21 dBm, which is the minimum among all the channels, whereas the receiver sensitivity of channel 3 was around −18 dBm, which was the maximum we obtained. The receiver sensitivity of channel 2 was equal to −19 dBm. The reason behind this variation in the receiver sensitivities of the different channels is the variation in the gain for the different signal wavelengths, as shown in Figure 3a.

**Figure 8:**
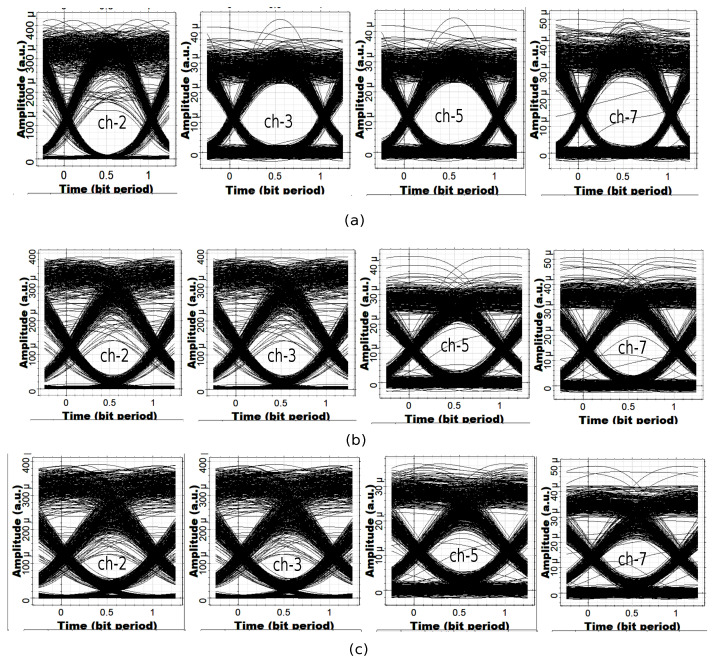
Eye diagrams of channels 2, 3, 5, and 7 at (**a**) 5 Gbps, (**b**) 7.5 Gbps, (**c**) 10 Gbps.

In telecommunication systems, an eye diagram is obtained by overlapping the bit periods of the signal over each other on the oscilloscope to obtain a plot of the signal amplitude with respect to time [33]. Since the shape of the resulting plot is similar to an eye, therefore, the name eye diagram is generally used [33]. Eye diagrams instantly provide visual information to a telecommunication system designer to check the received signal quality and predict the system BER [33]. There are two types of noise that can impact system performance, which are amplitude noise and timing jitter. Therefore, the eye diagram is another important alternative to BER measurements, which readily measure the extent of the amplitude noise and timing jitter in received signals. To further evaluate the performance of the channels, the eye diagrams of channels 2, 3, 5, and 7 were obtained at the output of the LPF by considering data rates of 5 Gbps, 7.5 Gbps, and 10 Gbps, as shown in Figure 8. As the data rate increased, the opening of the eye diagrams of channels 2, 3, 5, and 7 decreased due to the intensity fluctuation, timing jitter, and intersymbol interference (ISI). However, the eye diagrams of the channels had enough openings for the easy detection of ones and zeros [2].

## 5. Conclusions

A wideband and flat-gain hybrid optical amplifier operating in the O+E-band is proposed. The amplifier employs a parallel combination of a praseodymium-doped fiber amplifier and an SOA. The main problem of separating the O- and E-band signals to input them separately into the praseodymium-doped fiber amplifier and SOA has been solved using a wideband fiber Bragg grating. Moreover, various parameters, such as the PDF length, Pr${}^{3+}$ concentration, pump wavelength, and injection current of the SOA, have been precisely optimized to achieve the broadband amplification of a hybrid optical amplifier in the O+E-band with maximum flatness. The performance of the hybrid amplifier has been analyzed using the optimized parameters. A flat average gain and output power of 24 dB and 9.6 dBm, respectively, is achieved over the O+E-band. Noise figure in the range of 4–5.9 dB over a 1270–1450 nm wavelength region has been observed. The effect of up-conversion on the gain and output power of the hybrid amplifier is also investigated. The system-level performance of the hybrid amplifier has been analyzed as a pre-amplifier for a CWDM transmission system of eight OOK-modulated optical signals with an aggregate data rate of 80 Gbps.

## Figures and Tables

**Table 1:** Literature review of HOAs.

Study	Technology	Operating Band	Gain Bandwidth	Gain Flatness	NF
[13]	HBEDF-ZEDF	C+L	70 nm	1.8 dB	4.3–7.9 dB
[18]	SOA-EDFA-Raman	L	9 nm	0.8 dB	5–5.9 dB
[19]	EYDWA-Raman	C	23 dB	2.78 dB	5–6 dB
[20]	EYDFA-Raman	C	20 nm	1.37 dB	5.5–6 dB
[21]	Raman-EDFA	C+L	70 nm	-	3 dB
[22]	HDFA-TDFA	2000 nm	89 nm	-	6.5–7.5 dB
[24]	BDFA-EDFA	E+S	90 nm	-	7 dB
[25]	EDFA-OPA	S	60 nm	1.2 dB	5.1–6.1 dB
[26]	EDFA-Raman	C+L	-	1.9 dB	-
Proposed	PDFA-SOA	O+E	180 nm	1 dB	4–5.9 dB

**Table 2:** Symbols used in Equations (1)–(12) and their descriptions.

Symbol	Description
$n_{1}$, $n_{2}$	Ion densities at ground and excited manifolds
$n_{t}$	Total number of ions
$I_{p}$, $I_{s}$	Intensities of pump and signal
$P_{p}$, $P_{s}$	Power of pump and signal
$P_{p}\left(0\right)$, $P_{p}\left(z\right)$	Power of pump at input and a position z along the PDF
$P_{sat}$	Pump saturation power
$P_{a}$	Power absorbed
$R_{ij}$, $W_{ij}$	Transition rates of pump and signal between the *i*^th^ and *j*^th^ levels
$A_{ij}$	Rate of spontaneous emission between the *i*^th^ and *j*^th^ levels
$h\upsilon_{p}$, $h\upsilon_{s}$	Photon energies for pump and signal
$\sigma_{ap}$, $\sigma_{ep}$	Pump absorption and emission cross-sections
$\sigma_{as}$, $\sigma_{es}$	Signal absorption and emission cross-sections
$\sigma_{al}$, $\sigma_{el}$	Pump and signal absorption and emission cross-sections for length *l*
$\phi_{p}$	Quantum efficiency of pump
τ	Lifetime of the metastable state
*L*	PDF length
*A*	Effective area of fiber core
α	Effective loss coefficient
Γ	Optical confinement factor
*R*(*n*)	Recombinition rate
*I*, $I_{sp}$	Intensities of signal and spontaneous emission
*e*	Electron charge
*E*	Photon energy
β	Spontaneous emission coefficient
*V*	Active volume
*c*	Light velocity
$N_{m}$	Material index

**Table 3:** Main simulation parameters and their values.

Parameter	Value
Pump wavelength	1030 nm
Pump power	500 mW
PDF length	15.7 m
Pr${}^{3+}$ concentration	$50\times 10^{24}$ m${}^{-3}$
Core radius of PDF	1.2 μm
Doping radius of PDF	0.8 μm
Attenuation of SMF	0.2 dB/km
Numerical aperture of PDF	0.26
Center wavelength of WFBG	1310 nm
Bandwidth of WFBG	100 nm
Reflection of WFGB	99%
Injection current	60 mA
Length of SOA	$5\times 10^{-4}$ m
Width of SOA	$3\times 10^{-6}$ m
Height of SOA	$80\times 10^{-9}$ m
Optical confinement factor	0.3
Differential gain of SOA	$27.8\times 10^{-21}$ m${}^{2}$
Carrier density at transparency	$1.4\times 10^{24}$ m${}^{3}$
Initial carrier density	$30\times 10^{24}$ m${}^{-3}$
Signal attenuation	0.1 dB
Pump attenuation	0.15 dB
Temperature	300 K
Responsivity of PIN	0.9 A/W

## Data Availability

Not applicable.

## Abbreviations

The following abbreviations are used in this manuscript:

HOA	hybrid optical amplifier
BDFA	bismuth-doped fiber amplifier
SOA	semiconductor optical amplifier
NF	noise figure
PDFA	praseodymium-doped fiber amplifier
EDFA	erbium-doped fiber amplifier
CW	continuous wave
PDF	praseodymium-doped fiber
SMF	single-mode fiber
WFBG	wideband fiber Bragg grating
CWDM	coarse wavelength-division multiplexing
SDM	spatial division multiplexing
OMBT	optical multiband transmission
ASE	amplified spontaneous emission
GVD	group velocity dispersion
OPM	optical power meter
OSA	optical spectrum analyzer
OSNR	optical signal-to-noise ratio
ISI	intersymbol interference
BER	bit-error rate
